# Myocardial Alternative RNA Splicing and Gene Expression Profiling in Early Stage Hypoplastic Left Heart Syndrome

**DOI:** 10.1371/journal.pone.0029784

**Published:** 2012-01-27

**Authors:** Marco Ricci, Yanji Xu, Harriet L. Hammond, David A. Willoughby, Lubov Nathanson, Maria M. Rodriguez, Matteo Vatta, Steven E. Lipshultz, Joy Lincoln

**Affiliations:** 1 Division of Cardiothoracic Surgery, University of Miami Miller School of Medicine and Holtz Children's Hospital/Jackson Memorial Hospital, Miami, Florida, United States of America; 2 Shaun and Lilly International, LLC, Palm City, Florida, United States of America; 3 Center for Cardiovascular and Pulmonary Research, Nationwide Children's Hospital, Department of Pediatrics, The Ohio State University, Columbus, Ohio, United States of America; 4 Ocean Ridge Biosciences LLC, Palm Beach Gardens, Florida, United States of America; 5 Institute for Human Genomics, University of Miami Miller School of Medicine, Miami, Florida, United States of America; 6 Department of Pathology, University of Miami Miller School of Medicine and Holtz Children's Hospital, Miami, Florida, United States of America; 7 Division of Pediatric Cardiology, Texas Children's Hospital/Baylor College of Medicine, Houston, Texas, United States of America; 8 Department of Molecular Physiology and Biophysics, Baylor College of Medicine, Houston, Texas, United States of America; 9 Department of Pediatrics, University of Miami Miller School of Medicine and Holtz Children's Hospital, Miami, Florida, United States of America; I2MC INSERM UMR U1048, France

## Abstract

Hypoplastic Left Heart Syndrome (HLHS) is a congenital defect characterized by underdevelopment of the left ventricle and pathological compensation of the right ventricle. If untreated, HLHS is invariably lethal due to the extensive increase in right ventricular workload and eventual failure. Despite the clinical significance, little is known about the molecular pathobiological state of HLHS. Splicing of mRNA transcripts is an important regulatory mechanism of gene expression. Tissue specific alterations of this process have been associated with several cardiac diseases, however, transcriptional signature profiles related to HLHS are unknown. In this study, we performed genome-wide exon array analysis to determine differentially expressed genes and alternatively spliced transcripts in the right ventricle (RV) of six neonates with HLHS, compared to the RV and left ventricle (LV) from non-diseased control subjects. In HLHS, over 180 genes were differentially expressed and 1800 were differentially spliced, leading to changes in a variety of biological processes involving cell metabolism, cytoskeleton, and cell adherence. Additional hierarchical clustering analysis revealed that differential gene expression and mRNA splicing patterns identified in HLHS are unique compared to non-diseased tissue. Our findings suggest that gene expression and mRNA splicing are broadly dysregulated in the RV myocardium of HLHS neonates. In addition, our analysis identified transcriptome profiles representative of molecular biomarkers of HLHS that could be used in the future for diagnostic and prognostic stratification to improve patient outcome.

## Introduction

In Hypoplastic Left Heart Syndrome (HLHS), the right ventricle (RV) is exposed to pressure overload, volume overload, and hypoxia.[1], [2] Despite recent medical advances, RV failure remains the leading cause of death in children with HLHS.[3] Recent studies have identified HLHS as a heritable phenotype associated with certain chromosomal regions,[4], [5] however, to our knowledge, the molecular pathobiological state of HLHS has not been examined. Identifying biomarkers of disease state and progression is important for prognostic and therapeutic purposes to ultimately improve patient outcome.

Alterations in mRNA expression and splicing have previously been reported in a wide range of adult cardiac disease states,[6], [7] and affected transcripts have been considered as biomarkers of adult cardiomyopathies and heart failure.[8], [9] Alternative mRNA splicing is an important mechanism for generating transcriptional diversity and regulating gene expression in specific tissues, including the myocardium.[10] An increasing number of human disease states have been attributed to alterations in mRNA splicing as a result of genetic mutations and/or environmental causes.[11] Further, the development of global exon level interrogation techniques have provided insights into the pathobiology of these disease processes at the level of cell- and tissue-specific mRNA splicing events.[12], [13] Despite emerging studies examining transcriptome profiles in adult cardiac disease, the mRNA events associated with congenital heart disease (CHD) are less well understood. Genetic studies have suggested a possible role of mRNA alternative splicing in the pathogenesis of certain types of CHD.[14], [15] However to our knowledge, genome-wide mRNA expression and splicing profiling has not been utilized to investigate the myocardial pathobiology of HLHS.

The objective of this study was to characterize mRNA expression and splicing patterns in the RV myocardium of HLHS newborns by utilizing genome-wide exon-level interrogation, hypothesizing that mRNA expression and splicing profiles are dysregulated in the HLHS myocardium as compared to control LV and RV myocardium. As RV failure is the strongest predictor of long-term outcome in HLHS, our study focused on defining the gene expression and splicing patterns in the HLHS right ventricular myocardium to provide insights into the molecular mechanisms associated with RV adaptation or remodeling. In addition, as previous work by our group has shown that TGF-β-associated gene expression profiles in the HLHS-RV share greater molecular similarities with Control-LV than Control-RV,[16] we included HLHS-RV versus Control-LV comparisons in our analysis. Using this approach, our analyses revealed previously unappreciated insights into the molecular pathobiological state of early stage HLHS. The findings from this study provide candidate genes for the development of therapeutic assays that could improve prognostic stratification and clinical management of children with HLHS.

## Methods

### Tissue collection

This study was approved by the Institutional Review Board at the University of Miami (Protocol #20101106). For this study, tissue samples were collected from three experimental groups as follows: 1) six RV samples from neonates with HLHS (HLHS-RV), 2) five RV samples from control neonates (control-RV), and 3) five LV samples from the same control neonates (control-LV). As previously reported,[16] RV myocardial samples were obtained at the time of surgery from six neonates with HLHS undergoing Stage 1 Norwood reconstruction (See Results and Table 1). Myocardial sampling was carried out after institution of cardiopulmonary bypass and prior to induction of cardioplegic arrest. The diagnosis of HLHS was defined as previously reported by others.[3] Myocardial tissue from non-diseased controls was obtained at autopsy from the left ventricle (LV) and right ventricle (RV) of five newborn with normal cardiac anatomy, as previously described by our group[16] and others.[17] ^-18^ Autopsy specimens were collected by the same operator and absence of heart valve pathology was confirmed during post-mortem examination. Consistent with HLHS tissue harvesting, full-thickness RV tissue was obtained from the same area of the RV free wall in controls, whereas LV tissue was obtained from the mid-portion of the free wall. In each experimental group, approximately 50 mg of ventricular tissue were removed, snap frozen in liquid nitrogen, and stored at −80°C until RNA extraction.

**Table 1:** Preoperative clinical and echocardiographic characteristics of six newborns with Hypoplastic Left Heart Syndrome.

**Age, mean (range), days**	5 (2 to 7)
**Gestational Age, mean (range), weeks**	38 (36–40)
**Sex, male/female**	3/3
**Echocardiographic Data:**	**n = 6 neonates**
Ascending Aortic Size, mean (range), mm	2.2 (1.7–3.9)
Aortic Atresia (AA) vs. Stenosis (AS)	
AA	5
AS	1
Mitral Atresia (MA) vs. Stenosis (MS)	
MA	5
MS	1
Tricuspid Regurgitation	
None	1
Trivial	2
Mild	3
RV Function*	4
Normal	2
Mildly reduced	
Atrial Septum	
Non-obstructed	5
Mild obstruction	1
Ductus Arteriosus	
Non-obstructed	6
Obstructed	0
*RV function was defined qualitatively	

### RNA preparation and processing

RNA was extracted using TRIzol reagent (Invitrogen) or Qiagen RNeasy Mini Kit according to the manufacturer's instructions. In all samples, RNA integrity and purity were determined with the Agilent 2100 Bioanalyzer (Agilent Technologies, Inc., Santa Clara, CA). For microarray analysis, RNA samples were amplified and labeled using NuGEN Ovation Pico WTA system, WT-Ovation Exon module and Encore Biotin Module (NuGEN, San Carlos, CA). Briefly 20 to 50ng of total RNA were subjected to the synthesis of first-strand cDNA using a unique DNA/RNA chimeric primer mix and reverse transcriptase. DNA-RNA heteroduplex double-strand cDNA was generated and subjected to SPIA amplification, using an SPIA DNA/RNA chimeric primer, DNA polymerase and RNase H in a homogeneous isothermal assay. The resulting cDNA was used to generate sense transcript cDNA suitable for fragmentation and labeling as target for Affymetrix Human Exon 1.0 ST arrays.

### Hybridization of HLHS exon arrays

Microarrays (Affymetrix Human Exon 1.0 ST Arrays)[18] were hybridized for 17 hours at 45^o^C in the Affymetrix Hybridization Oven 645 according to the manufacturer's instructions. After hybridization, microarrays were washed, stained using the Affymetrix Fluidics Station 450, and scanned with the Affymetrix Scanner 3000 7G.

### Analysis of microarray data

Images were analyzed with the Affymetrix Command Console Software. The resulting CEL files were loaded into Exon Array Analyzer (http://eaa.mpi-bn.mpg.de/) and R respectively for quality control, and then processed by Affymetrix power tools (APT) for background correction, normalization, and summarizations with the Robust Multiple Average (RMA) algorithm to generate exon- and gene-level intensity estimates. The analysis was restricted to 287,329 exon-level and 22,011 gene-level core probe sets by selecting the core.ps and core.mps file options during exon- and gene-level processing with APT. The number of exon-level probe sets for consideration was further reduced by removing known cross-hybridizing exon-level probe-sets, per annotation file “HuEx-1_0-st-v2.na31.hg19.probeset.csv”.

#### Setting analysis thresholds

A custom ‘R’ script was then used to eliminate data for non-expressing genes and exons from the data set prior to hypothesis testing. In the first filtration step, exon-level probe sets were marked as detected when the Affymetrix-provided detection above background (DABG) p value was <0.05 in <50% of the samples of at least one of the three treatment groups. In the second filtration step, a transcript cluster was marked as detected if >50% of exon-level probe sets comprising the cluster were detected per filtration step 1.[19] All exon-level probe sets marked as undetected in step 1 and all exon-level probe sets making up undetected transcript clusters per step 2 were dropped to obtain a table of 166,628 detectable exon-level probe sets. The gene-level data table from the RMA output was then filtered to retain 13,628 transcript clusters mapping to one or more of the detectable exon-level probe sets. Some transcript clusters represented in the exon-level data were not represented in the gene-level RMA output, reducing the final list of detectable exon-level probe sets to 166,404.

The splicing index for each exon was calculated as: log_2_ (exon-level probe set intensity/gene-level probe set intensity). BioConductor LIMMA package was used for differential analysis. One-way ANOVA of splicing indexes and gene-level probe intensities was performed to identify exon-level and gene-level probe sets (i.e. transcript clusters) showing significant changes in intensity among the three treatment groups. T tests were additionally used for comparisons amongst two groups: HLHS-RV vs. Control-RV and HLHS-RV vs. Control-LV. The false discovery rate (FDR) for each P value was calculated by the method of Benjamini and Hochberg.[20]

#### Venn Diagrams

Genes corresponding to differentially expressed transcript clusters or exon probe sets with an altered splice index were selected for representation in Venn diagrams and further pathway analysis, providing threshold criteria were met (FDR of <0.05 in the three-group one way ANOVA, an FDR <0.05 in the t-test for the specific two group comparison, and an intensity or splice-index (exon probes) difference of >1.5-fold). WebGestalt Software (Vanderbilt University)[21] was used to identify KEGG pathways showing a statistically significant over-representation of genes with altered total mRNA or exon levels in the HLHS-RV samples compared to control groups. The 13,628 transcript clusters (genes) that showed detectable signal on the array were used as the reference set for calculating statistical significance in the pathway analysis. Venn Diagram Plotter was used to draw Venn diagrams to compare genes either with significant alternative splicing or significant overall expression changes within different groups.

#### Clustering analysis

Criteria for selection of differentially expressed transcript clusters or exon probe sets with an altered splice index for display in hierarchical clustering was the same as for the pathway analysis, other than a 2-fold change threshold in gene-level probe set intensity or a 5-fold change in splicing-index for exon-level probe sets were applied. Data for the detectable probes were clustered using Cluster 3.0 software.[22] The log_2_-transformed data were pre-processed by median centering of the data for each probe set, and then hierarchically clustered using centered correlation as the similarity metric and average linkage as clustering method.

#### Principal component analysis

Principal component analysis was conducted using Partek to visualize the overall pattern of gene expression. All data is MIAME-compliant (Minimum Information About a Microarray Experiment) and all CEL files for this microarray study are available through The Gene Expression Omnibus at: http://www.ncbi.nlm.nih.gov/geo/query/acc.cgi?token=hfapraoacwqgwhg&acc=GSE23959.

#### Quantitative real-time PCR analysis

To validate differential fold changes in gene expression between the comparison groups from the microarray analysis, cDNA was generated from 400 ng tRNA from human samples using the High Capacity Reverse Transcriptase kit (Applied Biosystems). Then, 10 µL cDNA were subjected to quantitative real-time PCR using a custom-designed Taqman Low Density Array (Applied Biosystems) containing two endogenous controls (*18s, GAPDH)* and 94 random genes that significantly changed in the microarray analysis as described.^21^ Cycle counts for each target gene were normalized to *18s* expression, and significant differences in gene expression were reported as a fold change compared to each experimental group.^21^

## Results

### Myocardial gene expression and mRNA splicing are altered in HLHS

To determine global transcriptome changes in early stage HLHS pathogenesis, gene expression and exon array profiling (Affymetrix Human Exon 1.0 ST Array) was performed using total RNA isolated from the RV myocardium of neonates with HLHS (n = 6) (HLHS-RV), and compared to RNA collected from control-RV (n = 5), and control-LV (n = 5) samples. For each RNA sample collected, the analysis was performed using the Agilent Bio-analyzer 2100 to confirm consistently high levels of RNA integrity and purity across the sample groups. The clinical and echocardiographic data of the six HLHS neonates included in this study are shown in Table 1. To facilitate the interpretation of data, neonates with extra-cardiac anomalies or known chromosomal defects were excluded from the HLHS group. All 6 HLHS subjects were in a relatively compensated hemodynamic state, had normal acid-base balance, and received Prostaglandin E1 infusions before undergoing a Stage 1 Norwood procedure. The mean gestational age of HLHS neonates was 38 weeks (range 36–40) and the mean postnatal age was 5 days. For controls, the five subjects expired from various non-cardiac disease processes including intra-ventricular hemorrhage, meconium aspiration, and necrotizing enterocolitis. The mean gestational age of control subjects was 33 weeks (range 26 to 39 weeks), and the mean postnatal age was 18 days.

**Figure 1:**
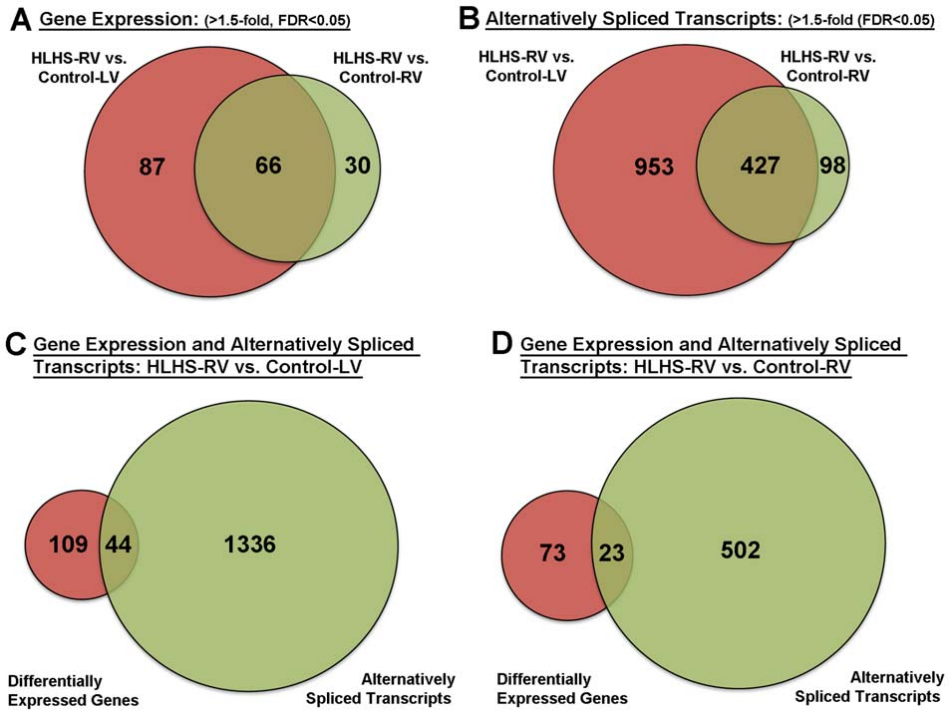
Venn diagrams showing the number of differentially expressed genes (A) and alternatively spliced transcripts (B) common and unique in HLHS vs. Control-LV (red) and HLHS vs. Control-RV (green) sample groups. (C, D) Venn diagrams to indicate the number of transcripts that were differentially expressed (red) and/or alternatively spliced (green) in HLHS-RV vs. Control-LV (C) and HLHS vs. Control-RV (D) sample groups. Fold change in gene expression and splicing index >1.5-, FDR, False Discovery Rate <0.05.

In this study, RNA was hybridized to Affymetrix arrays containing approximately 40 probes per gene and 4 probes per exon, therefore allowing two complementary levels of analysis in the same samples at the levels of gene expression and alternative splicing, respectively. One-way ANOVA analysis was performed to compare ‘core’ gene (17,800 transcript clusters: Refseq and full length GenBank mRNAs) expression and alternative splicing profiles in HLHS-RV tissue vs. Control-LV, and Control-RV samples. Significant changes were considered with fold changes in gene expression and splicing index >1.5 and a false discovery rate (FDR) of <0.05. Using these parameters to compare HLHS-RV to control samples, a total of 183 genes were differentially expressed: 153 genes in HLHS-RV vs. Control-LV, 96 genes when compared to Control-RV, with 66 genes overlapping between the two comparison groups (Figure 1A). Tables 2, 3, 4, 5 show the top ten most differentially expressed genes and alternatively spliced transcripts in the HLHS-RV when compared to each control group (Control-RV and Control-LV). Using exon array analysis, we identified 1478 genes affected by alternative splicing in HLHS-RV samples compared to control groups. Of the 1380 spliced genes in HLHS-RV vs. Control-LV, only 44 (3%) showed significant changes in gene expression. The number of alternatively spliced transcripts was much lower in HLHS-RV when compared to Control-RV (525 genes), although the percentage of genes that were both spliced and differentially expressed was similar (23 transcripts, 4%). Worthy of mention, 13 of these transcripts were common to both HLHS-RV vs. Control-LV and HLHS-RV vs. Control-RV comparative analyses. Table S1 lists all common and unique differentially expressed genes and alternatively spliced transcripts within each analysis group as indicated in Figure 1.

**Table 2:** Top ten most differentially expressed genes and alternatively spliced transcripts in the HLHS-RV group: HLHS-RV vs. Control-LV (Differential Gene Expression).

Gene Symbol	HLHS-RV vs. Control-LV Fold Change	HLHS-RV vs. Control-LV p-value	HLHS-RV vs. Control-LV FDR	HLHS-RV vs. Control-RV Fold Change	HLHS-RV vs. Control-RV p-value	HLHS-RV vs. Control-RV FDR
*FAM129A*	2.1	3.3E-07	2.3E-03	2.0	7.2E-07	2.2E-03
*LANCL2*	2.1	1.9E-07	2.3E-03	1.8	2.6E-06	3.0E-03
*RGN*	2.3	5.9E-07	2.7E-03	2.5	1.5E-07	1.5E-03
*RAB3IP*	2.2	1.7E-04	2.4E-02	3.2	2.1E-06	2.7E-03
*MRPL46*	2.5	7.7E-06	1.0E-02	2.3	2.4E-05	1.4E-02
*C21orf62*	2.9	9.7E-06	1.0E-02	2.8	1.3E-05	9.4E-03
*IFI44*	2.7	4.8E-06	1.0E-02	2.1	1.1E-04	2.9E-02
*BRP44L*	2.4	8.8E-06	1.0E-02	2.2	3.1E-05	1.4E-02
*DCTN3*	2.5	5.6E-06	1.0E-02	1.9	4.3E-04	4.6E-02
*HBB*	0.3	2.5E-05	1.6E-02	0.3	2.6E-05	1.4E-02

**Table 3:** Top ten most differentially expressed genes and alternatively spliced transcripts in the HLHS-RV group: HLHS-RV vs. Control-RV (Differential Gene Expression).

Gene Symbol	HLHS-RV vs. Control-LV Fold Change	HLHS-RV vs. Control-LV p-value	HLHS-RV vs. Control-LV FDR	HLHS-RV vs. Control-RV Fold Change	HLHS-RV vs. Control-RV p-value	HLHS-RV vs. Control-RV FDR
*FAM129A*	2.1	3.3E-07	2.3E-03	2.0	7.2E-07	2.2E-03
*RGN*	2.3	5.9E-07	2.7E-03	2.5	1.5E-07	1.5E-03
*USP2*	1.9	6.4E-06	1.0E-02	2.3	2.2E-07	1.5E-03
*AIFM1*	1.8	8.8E-06	1.0E-02	2.0	1.1E-06	2.2E-03
*PSD3*	1.9	6.4E-05	1.9E-02	2.5	6.7E-07	2.2E-03
*COQ10A*	1.9	1.3E-04	2.3E-02	2.6	1.8E-06	2.7E-03
*COL6A3*	0.6	1.3E-03	5.2E-02	0.4	1.9E-06	2.7E-03
*RAB3IP*	2.2	1.7E-04	2.4E-02	3.2	2.1E-06	2.7E-03
*MRPL46*	2.5	7.7E-06	1.0E-02	2.3	2.4E-05	1.4E-02
*C21orf62*	2.9	9.7E-06	1.0E-02	2.8	1.3E-05	9.4E-03

**Table 4:** Top ten most differentially expressed genes and alternatively spliced transcripts in the HLHS-RV group: HLHS-RV vs. Control-LV (Alternatively Spliced Transcripts).

Gene Symbol	HLHS-RV vs. Control-LV Fold Change	HLHS-RV vs. Control-LV p-value	HLHS-RV vs. Control-LV FDR	HLHS-RV vs. Control-RV Fold Change	HLHS-RV vs. Control-RV p-value	HLHS-RV vs. Control-RV FDR
*INADL*	5.38	1.5E-02	4.91	1.2E-04	4.1E-02	1.5E-02
*SPTA1*	0.16	6.4E-03	0.13	2.0E-06	6.5E-03	6.4E-03
*ENAH*	0.16	2.2E-08	4.0E-04	0.21	2.1E-07	2.4E-03
*RYR2*	6.02	1.6E-05	7.8E-03	3.47	6.9E-04	8.0E-02
*ITGB1BP1*	5.53	6.4E-07	1.9E-03	2.95	1.4E-04	4.3E-02
*IFT172*	0.18	1.2E-04	1.8E-02	0.43	2.5E-02	3.0E-01
*C2orf55*	5.24	5.6E-04	3.6E-02	5.68	3.6E-04	6.2E-02
*GYPB/GYPA*	0.16	7.3E-07	1.9E-03	0.17	1.0E-06	4.4E-03
*CPNE5*	0.16	4.0E-05	1.1E-02	0.44	2.4E-02	3.0E-01
*C6orf186/DDO*	6.49	2.8E-06	3.8E-03	2.91	1.1E-03	9.4E-02

**Table 5:** Top ten most differentially expressed genes and alternatively spliced transcripts in the HLHS-RV group: HLHS-RV vs. Control-RV (Alternatively Spliced Transcripts).

Gene Symbol	HLHS-RV vs. Control-LV Fold Change	HLHS-RV vs. Control-LV p-value	HLHS-RV vs. Control-LV FDR	HLHS-RV vs. Control-RV Fold Change	HLHS-RV vs. Control-RV p-value	HLHS-RV vs. Control-RV FDR
*SPTA1*	0.05	2.0E-09	8.4E-05	0.07	9.9E-09	4.2E-04
*MXD1*	0.21	4.2E-05	1.2E-02	0.17	6.8E-06	1.1E-02
*GYPB/GYPA*	0.16	7.3E-07	1.9E-03	0.17	1.0E-06	4.4E-03
*GRM1*	4.84	7.4E-05	1.5E-02	5.04	5.7E-05	2.9E-02
*ANK1*	3.60	6.3E-04	3.8E-02	6.18	1.7E-05	1.6E-02
*CACNB2*	0.17	2.1E-05	8.8E-03	0.18	3.2E-05	2.2E-02
*CCNJ*	0.17	5.5E-10	5.8E-05	0.19	1.1E-09	1.8E-04
*PPFIA2*	4.00	1.9E-04	2.2E-02	5.43	2.2E-05	1.9E-02
*PMP22*	2.40	2.0E-03	6.6E-02	5.89	1.1E-06	4.6E-03
*PLD6*	2.16	4.4E-02	2.8E-01	5.84	1.1E-04	4.0E-02

**Figure 2:**
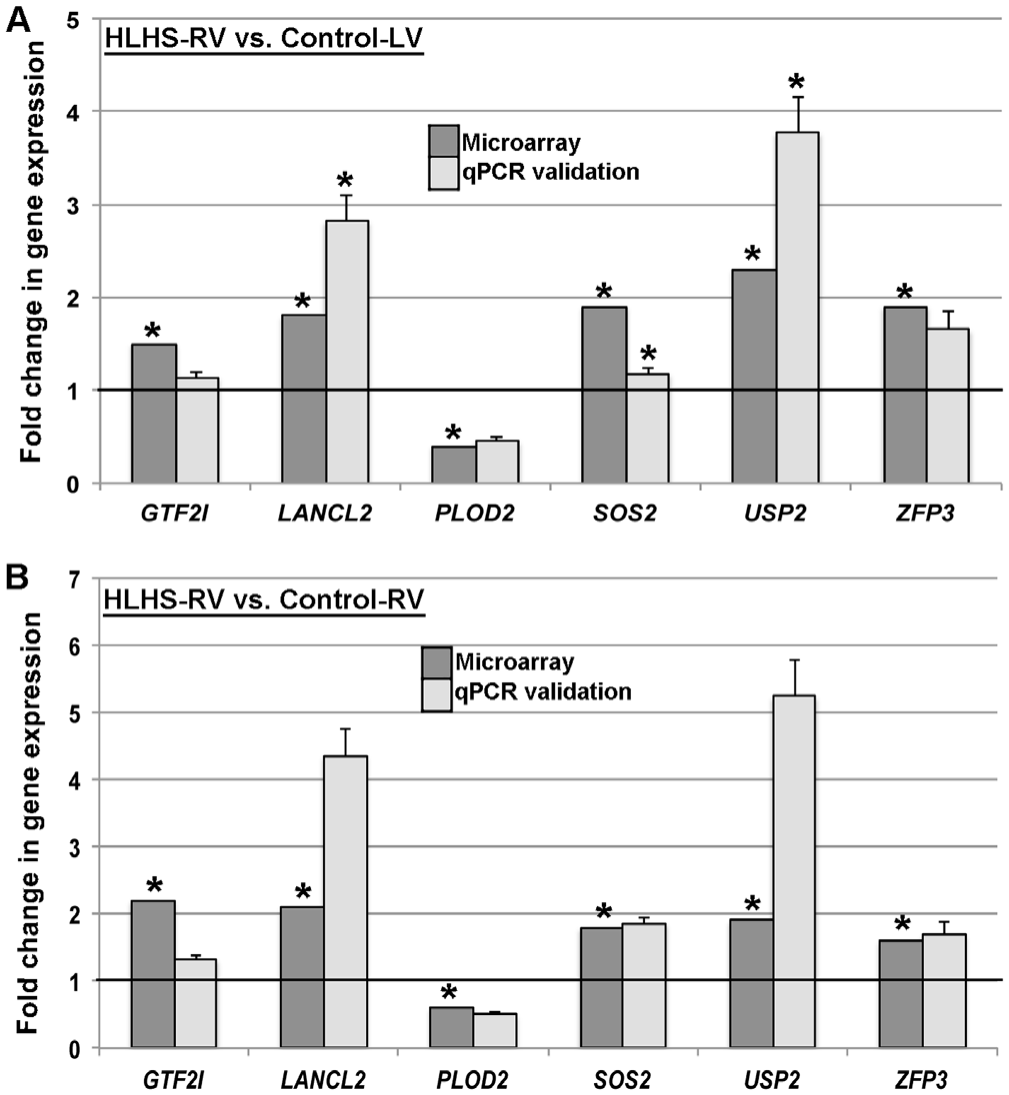
Validation of the microarray data by real-time qPCR on randomly selected genes. Dark grey bars indicate microarray findings and light grey bars indicate qPCR results. Error bars show SEMs. * p<0.05 in HLHS-RV compared to indicated control groups.

To confirm observations from the microarray data, a total of 94 randomly selected genes that passed the threshold criteria were validated by quantitative real-time PCR in n = 4 samples from each subject group (HLHS-RV, Control-LV, and Control-RV). Of the 94 genes, more than 86% showed consistent differential changes in gene expression in HLHS-RV samples when compared to the respective control groups. Examples of 6 validated genes (*GTF2I, LANCL2, PLOD2, SOS2, USP2* and *ZFP3*) are shown in Figure 2. Collectively, this analysis validates the significance of the microarray data and confirms the differential gene expression observed in HLHS-RV vs. controls groups.

### Clustering analysis distinguishes HLHS-RV samples from controls

**Figure 3:**
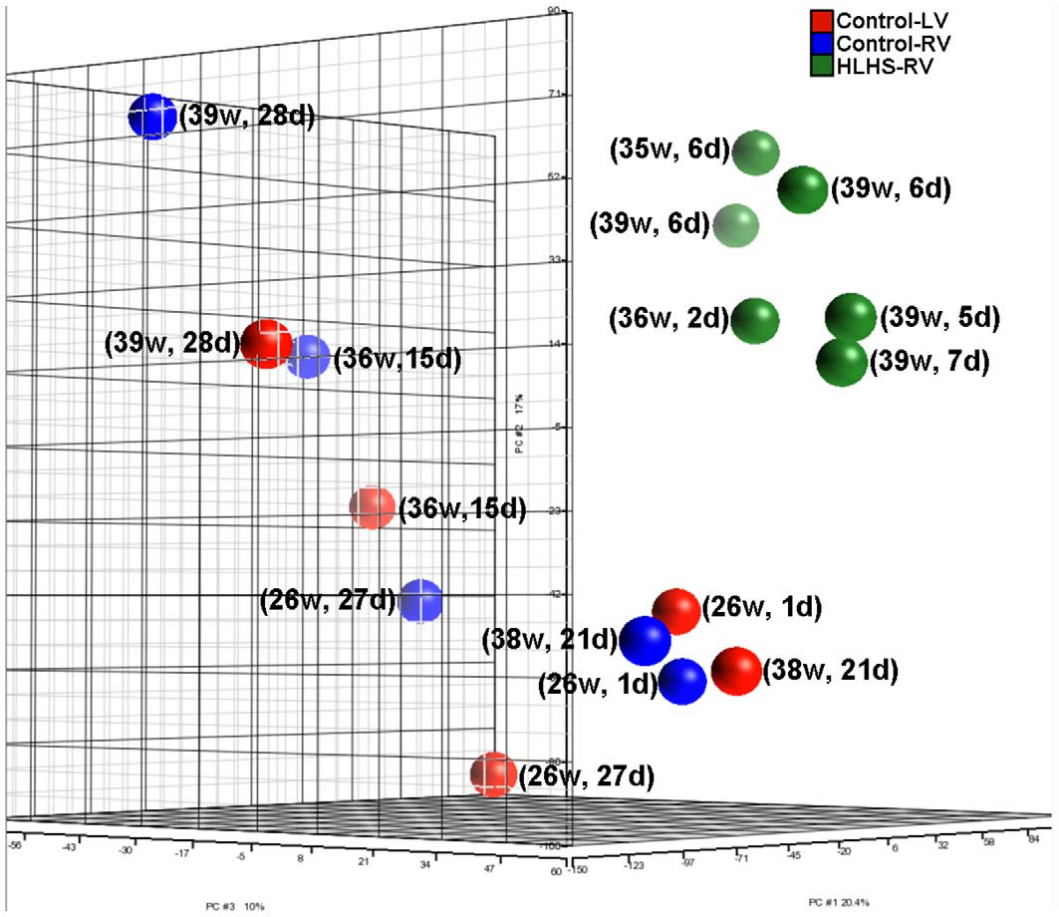
Principal component plot of normalized gene expression values from microarray analysis of six HLHS (green), five Control-LV (red), and five Control-RV (blue) samples. Gestational age (in weeks) and postnatal age (in days) of each subject is indicated in parentheses. HLHS-RV samples segregated apart from all Control-RV and Control-LV samples, irrespective of post-natal age and gestational age. Post-natal and gestational age did not appear to result in any particular trend either within the HLHS or the control groups.

To visually represent commonality or variance in the pattern of differentially expressed genes among the three sample groups, we generated a multidimensional principal component analysis (PCA) plot (Figure 3). This analysis illustrates similarities in transcriptome profiles within the six HLHS-RV samples (green), and a clear separation of profiles between HLHS-RV and the five Control-RV (blue) samples and Control-LV (red) samples (Figure 3). Importantly, the expression variance between HLHS-RV and control samples was maintained irrespective of postnatal age and gestational age, as transcriptional profiles between HLHS-RV and control samples never overlapped, even when samples were age-matched (Figure 3). This pattern of variance suggests that the differences in age between HLHS and control neonates unlikely contributed to the observed transcriptional variance between HLHS and controls. In addition, the PCA analysis revealed a modest transcriptional difference between Control-RV and Control-LV samples. However, within each control group there was substantial variability (Figure 3), likely reflecting the greater biodiversity and clinical heterogeneity of control subjects as compared to the more homogeneous HLHS group.

**Figure 4:**
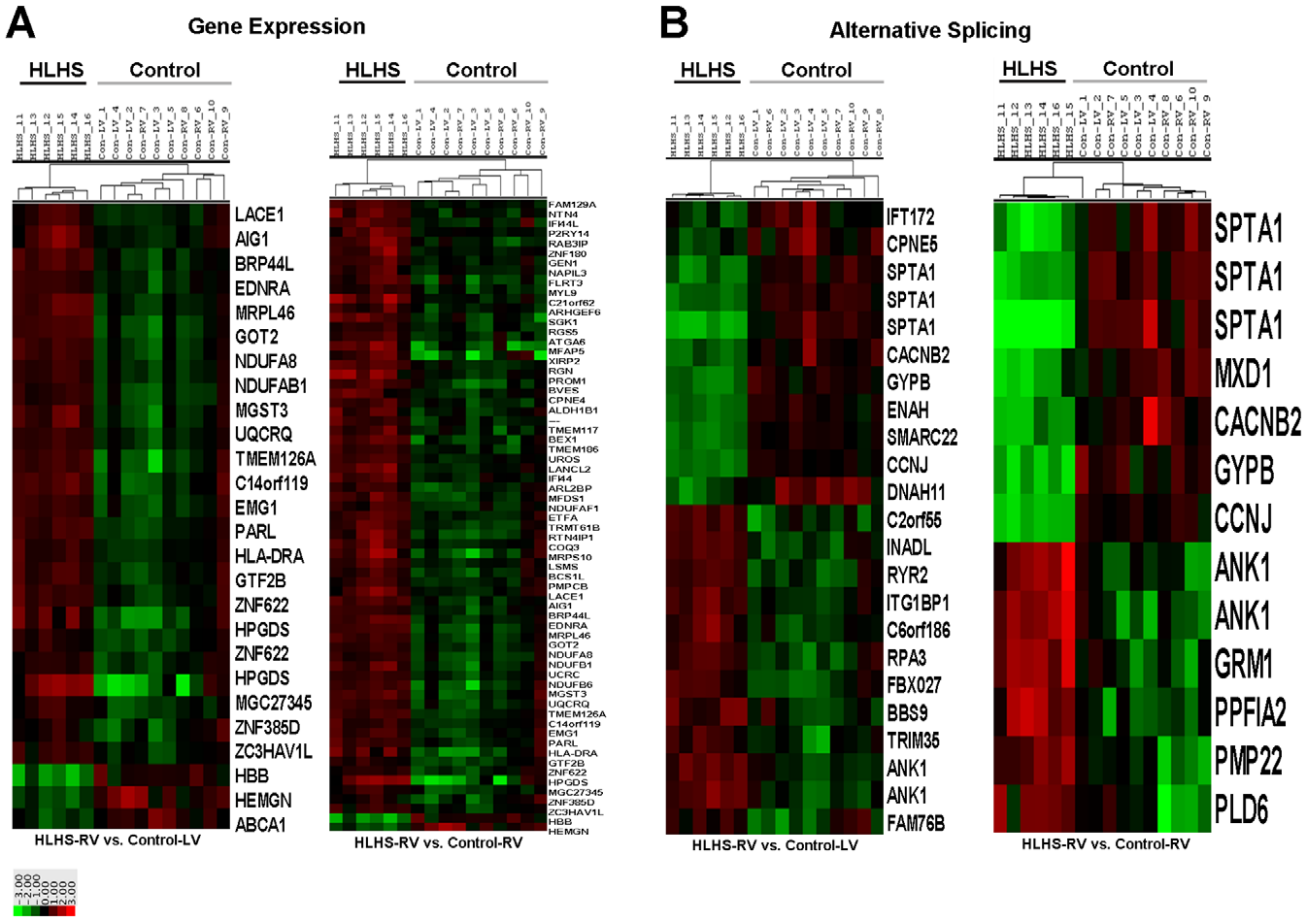
Hierarchical clustering and heat map analysis of differential gene expression (A) and alternatively spliced (B) profiles in HLHS-RV vs. Control-RV and Control-LV samples. Clustering was performed on log_2_-transformed and normalized gene-level probe set intensities and splicing indexes for transcript clusters or exon-level probe sets, respectively, that met specific significance and fold-change criteria (see Methods). Except for the right image of panel A, all of the probe sets meeting the specific criteria are displayed. Note significant clustering of HLHS samples compared to controls that show indistinguishable identity. Key: one unit = a difference of one log_2_ unit from the gene (A) or splicing index (B) mean for all the samples.

To further examine whether HLHS-RV gene expression and alternative splicing profiles segregated from control samples, hierarchical clustering analysis was performed. Figure 4 illustrates clustering analysis of differentially expressed genes (Figure 4A) and alternatively spliced transcripts (Figure 4B) in HLHS-RV vs. indicated controls. In both clustering analyses, the 6 HLHS-RV samples clustered separately from all control samples. Interestingly, hierarchical clustering of the 5 Control-LV and Control-RV samples was indistinguishable, suggesting indifferent gene expression and splicing profiles.

### Several biological processes are dysregulated in the HLHS myocardium

**Figure 5:**
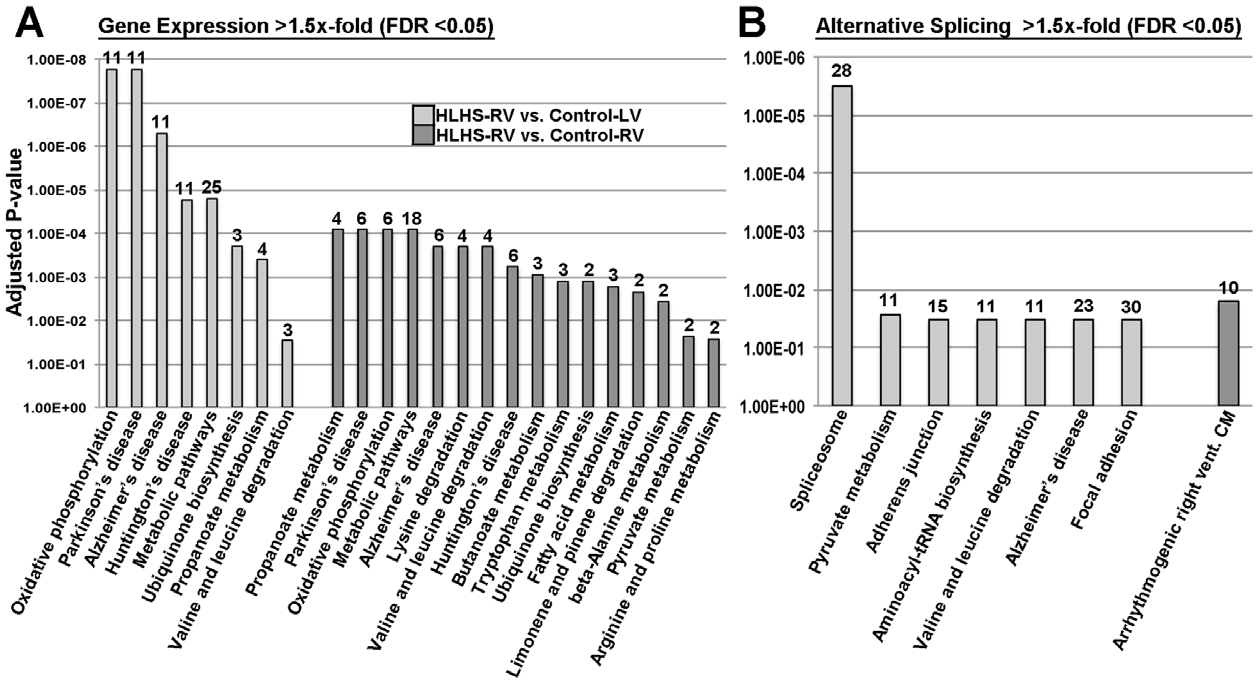
KEGG pathway analysis to show the relative significance of affected biological processes in HLHS-RV samples versus controls as a result of differential gene expression and alternative splicing events. Numbers indicate the number of differentially expressed or alternatively spliced transcripts that within each indicated KEGG category.

To determine the biological processes altered in the early stage HLHS-RV pathogenesis, KEGG pathway analysis was performed. Figure 5 shows the significantly affected KEGG pathways as a result of differential changes in gene expression (Figure 5A) and alternatively spliced transcripts (Figure 5B) in HLHS-RV samples vs. Control-LV (light grey bars) and vs. Control-RV (dark grey bars). At the gene expression level, ‘oxidative phosphorylation’ was most significantly altered (p = 1.75E-05) in HLHS-RV compared to Control-LV samples. This process, along with other cell metabolism activities including ‘propanoate metabolism’ were also significantly affected (p = 8.04E-05) in HLHS-RV when compared to Control-RV samples. At the level of mRNA splicing, ‘spliceosome’ and ‘arrhythmogenic right ventricle cardiomyopathy’ KEGG pathways were significantly altered in HLHS-RV compared to Control-LV and Control-RV samples, respectively. The differentially expressed genes and alternatively spliced transcripts associated with these altered KEGG pathways are included in Table 6. These findings suggest a broad dysregulation in mRNA expression and splicing patterns in the HLHS myocardium involving a variety of biological processes.

**Table 6:** Most significantly affected KEGG pathways by differentially expressed genes and alternatively spliced transcripts in the HLHS-RV group.

KEGG Pathway	Differentially Expressed Genes	Fold Change (HLHS-RV vs. Control-LV)	p-value	FDR	Fold Change (HLHS-RV vs. Control-RV)	p-value	FDR
Oxidative Phosphorylation:			1.75E-05				
	*NDUFV1*	1.7	4.4E-04	3.4E-02	1.7	3.8E-04	4.3E-02
	*NDUFS1///LOC100329109*	1.9	1.4E-04	2.4E-02	1.9	1.4E-04	3.2E-02
	*NDUFB6///DFFB*	2.8	1.9E-04	2.4E-02	2.2	1.8E-03	8.0E-02
	*UQCRQ*	3.5	1.8E-04	2.4E-02	2.0	1.7E-02	2.0E-01
	*UQCR10*	2.1	5.2E-04	3.6E-02	2.0	1.1E-03	6.8E-02
	*SEC31B*	1.8	3.2E-05	1.7E-02	1.9	1.1E-05	8.1E-03
	*NDUFAB1*	2.0	1.1E-04	2.1E-02	1.8	9.6E-04	6.5E-02
	*COX4I1*	1.6	3.3E-04	3.0E-02	1.5	9.2E-04	6.4E-02
	*ATP5A1*	1.6	9.6E-05	2.0E-02	1.7	2.7E-05	1.4E-02
	*SNORD59A///*	1.6	7.0E-05	1.9E-02	1.6	8.8E-05	2.8E-02
	*NDUFA8*	2.4	8.2E-05	2.0E-02	2.1	3.3E-04	4.2E-02
Propanoate Metabolism:						8.04E-05	
	*ACAT1///ACAT1*	1.9	9.2E-04	4.5E-02	2.1	4.1E-04	4.5E-02
	*PCCB//PCCB*	1.9	1.8E-04	2.4E-02	1.8	3.4E-04	4.2E-02
	*ALDH1B1*	2.1	5.9E-05	1.9E-02	2.0	2.0E-04	3.6E-02
	*ECHS1*	1.5	4.9E-04	3.5E-02	1.5	2.1E-04	3.6E-02

### Exon abundance of individual genes is altered in HLHS

**Figure 6:**
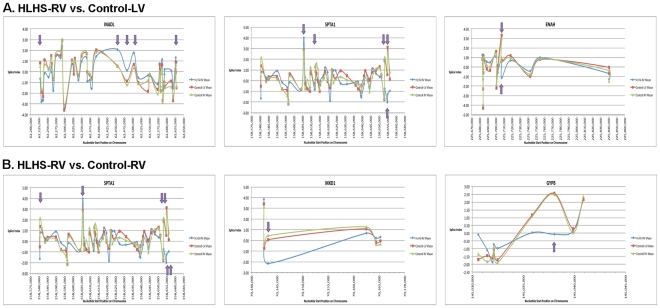
Splicing Index of the core probe set to indicate individual exon expression in the top three alternatively spiced transcripts in HLHS vs. Control-LV (A) and vs. Control-RV (B). Purple arrows indicate FDR<0.05 for HLHS vs. indicated control and one-way ANOVA.

To determine alterations in the abundance of individual exons of alternatively spliced genes in HLHS-RV, expression profiling was performed. Figure 6 shows the splicing index of pre-designed probe sets that hybridized at nucleotide positions consistent with individual exons of the top three transcripts affected my mRNA splicing (but not gene expression) in HLHS-RV samples compared to controls. As indicated by the arrows, HLHS-RV samples show distinct exon-specific expression profiles compared to control samples and provide insights into the post-transcriptional events active in early stage HLHS.

## Discussion

Recent studies have partly unveiled the chromosomal loci associated with hereditary HLHS,[4], [5] however the molecular pathobiology of this disease remains poorly understood. Recent studies have demonstrated significant changes in mRNA expression and splicing profiles in a variety of adult cardiac pathophysiologic states, although transcriptome alterations in CHD have not been reported.[12] To address this, we performed a genome-wide exon array and found significant changes in gene expression and alternative splicing events in the RV of a representative cohort of infants with HLHS compared to non-diseased controls. These transcriptome alterations were associated with changes in a variety of biological processes involving cell metabolism, the cytoskeleton, cell adherence and mRNA processing. Further, hierarchical clustering based upon gene expression and alternative splicing analysis data revealed unique molecular profiles segregating HLHS-RV samples from LV and RV control samples. Collectively, our findings provide new insights into the transcriptional events active in the RV myocardium of early stage HLHS. In addition, they provide the basis for the identification of novel transcriptome-based markers of disease state that may be useful for prognostic stratification and treatment purposes.

Signature profiles of gene expression have been used as tools for diagnostic and prognostic purposes in human cardiomyopathies.[23] In this study of congenital heart disease, a total of 183 core genes passed threshold criteria and were considered differentially expressed in HLHS-RV samples compared to controls. Among these, 66 genes overlapped between the two comparison groups (HLHS-RV vs. Control-LV and HLHS-RV vs. Control-RV). However, of the differentially expressed transcripts that were not common, the genetic profile of HLHS-RV was most similar to Control-RV. This observation is in contrast to our previous study showing that activity of TGF-β signaling pathways in HLHS-RV was most similar to Control-LV.[16] Despite the unique genetic profiles in HLHS-RV vs. Control-LV and Control-RV, differentially expressed genes associated with ‘oxidative phosphorylation’ were similarly affected in HLHS-RV when compared to each of the control groups (Table 6). Based upon the pathophysiological exposure of the RV to pressure overload, volume overload and hypoxia in HLHS, the overrepresentation of oxidative phosphorylation and other metabolic genes (Figure 5, Table 6) in the HLHS-RV myocardium suggest a shift from normal fatty acid metabolism to glucose metabolism found in myocytes undergoing hypertrophy. [24], [25] In addition, it is likely that the differential gene expression profile observed in HLHS-RV samples is representative of early post-natal adaptive RV remodeling in response to compensatory changes in myocardial hypertrophic growth. It is therefore surprising that other genes associated with myocardial hypertrophy were not differentially expressed including extracellular matrix genes and regulatory transcription factors. We suspect that this may be due to the collection of RNA from newborns with HLHS and not older patients in whom the RV remains exposed to pressure/volume overload and hypoxia for a longer period of time. However, more long-term studies are required to confirm this hypothesis.

In addition to altered whole-gene expression patterns, altered splicing patterns resulting in differential exon abundance in HLHS tissue are also consistent with other studies of adult cardiac disease states.[12] ^,^ [26] ^,^ [27] Similar to differential gene expression, cell metabolism processes were also significantly altered by mRNA splicing. However, KEGG pathway analysis revealed that splicing events also led to significant changes in transcripts associated with ‘spliceosome’ and ‘adherens junctions’ categories in HLHS-RV compared to Control-LV, and ‘arrhythmogenic right ventricle cardiomyopathy’ when compared to Control-RV. The individual transcripts affected within these broad KEGG categories included genes associated with mRNA processing (*Prp 2, 16–18, 53*), consistent with the overrepresentation of the ‘spliceosome’ and consistent with splicing events. In addition, genes involved with cell-cell communication (*CTNNB1, DSG2, ITGB3, CDH2*), cytoskeleton (*DMD, SGCG*), and calcium movement (*CACNA1C, CACNB2*) were largely underexpressed in HLHS-RV. These latter changes are consistent with compensatory remodeling in the RV of HLHS patients to support pathophysiological changes in hemodynamic load as a result of an underdeveloped LV. Although informative, we cannot determine if the observed changes in gene expression and mRNA splicing are causative of HLHS or the result of adaptive or maladaptive myocardial changes in the RV myocardium of HLHS subjects. Nonetheless, this analysis provides insights into the biological processes that are altered in the HLHS-RV as a result of changes in transcriptome profiles.

In this study we have identified a number of differentially expressed and differentially spliced transcripts that have the potential to serve as biomarkers of HLHS disease state. These include differential expression and splicing of calcium transporter (*SLC8A1, CACNB2, RYR1*) and energy metabolism (*COX4I1, ACAT1, ATP5A1*) genes, as well as structural proteins (*STPB, DCTN5, XIRP2*), detectable in myocardial samples obtained from HLHS patients during surgical intervention. In addition to tissue-specific biomarkers, levels of secreted factors (*IFI44, VEGFA*) and cell surface markers (*PROM1*) have previously shown to be measurable in serum samples and serve as biomarkers for other pathological states.[28] Our findings also revealed that, in HLHS, less than 5% of alternatively spliced transcripts are also differentially expressed. In addition, we have also observed that splicing events result in variable levels of individual exon abundance, suggesting that gene dosage may have important implications in understanding the mechanisms of HLHS and RV failure. Therefore, in the development of a prognostic and therapeutic HLHS biomarker assay, it is important to use exon interrogation and include splicing profiling to target specific chromosomal exon locations as an adjunct to gene expression analysis. As HLHS remains associated with a considerable risk of RV failure, this study holds promise as it establishes the basis for using mRNA expression-based and splicing-based profiling in myocardial tissue or even in serum samples for prognostic purposes and to guide therapeutic interventions.

We acknowledge that this study presents several limitations. While somewhat uncontrollable, it is recognized that compared to HLHS subjects, control tissue was obtained from a heterogeneous group of newborns aged <37 weeks of gestation who died from non-cardiac disease states. Although our PCA selective analysis (Fig. 3) suggests that pre-natal age was unlikely a major contributor to differential expression, it is possible that this factor introduced a bias in our study. Similarly, the unavoidable deterioration or stress in the control subjects during the period leading to their demise might have played a role. Unfortunately, sudden neonatal death due to non-cardiac causes is a very rare event, and therefore ideal control subjects are usually not available. Also, while our analysis provides a descriptive cross-sectional assessment of genomic profiling at a specific maturational age, it does not identify which pathways are active during fetal life or in subsequent developmental stages. Further work is required to determine transcriptome profiles in a larger cohort of HLHS subjects at different stages of disease state. Also, it is exceedingly difficult to obtain myocardial tissue from age-matched control living subjects and it is recognized that this might have introduced a bias, although RNA quality from post-mortem samples was consistent with that from HLHS subjects. Finally, our approach is unable to distinguish between mRNA splicing events causative of HLHS phenotypes and those that are secondary to pathophysiology-induced alterations.

In conclusion, using genome-wide exon interrogation we have identified new mRNA expression and splicing signature profiles associated with early stage HLHS pathobiology. Our findings suggest that mRNA expression and splicing are broadly dysregulated in the RV myocardium of HLHS neonates. This study provides novel clues into the molecular events and biological processes associated with neonatal HLHS pathobiology. In addition, this dataset establishes the basis for future investigations to identify transcriptome-based biomarkers of disease severity and progression that could be useful for diagnostic and prognostic stratification to improve patient outcome.

## Supporting Information

Table S1 **Summary Table to show the list of differentially expressed and alternatively spliced transcripts in HLHS-RV vs. Control-LV and Control-RV samples groups.** Transcripts are ranked in order of chromosome location.
(XLSX)
